# The cholera outbreak in Lahore, Pakistan: challenges, efforts and recommendations

**DOI:** 10.1186/s41182-022-00458-9

**Published:** 2022-09-02

**Authors:** Aiman Naveed, Mohammad Umer, Muhammad Ehsan, Muhammad Ayyan, Abia Shahid, Afra Zahid, Mohammad Yasir Essar, Huzaifa Ahmad Cheema

**Affiliations:** 1 Department of Medicine, King Edward Medical University, Lahore, Pakistan; 2 Kabul University of Medical Sciences, Kabul, Afghanistan; 3 Department of Community Medicine, King Edward Medical University, Lahore, Pakistan

**Keywords:** Cholera, Lahore, Punjab, *Vibrio cholerae*, Healthcare

## Abstract

The Punjab health authorities have declared a cholera outbreak with over 2000 acute diarrheal cases being reported in Lahore in April 2022 and 25 laboratory-confirmed cases as of 27 May 2022. Overpopulation, poor sanitation, and a substandard water drainage system contribute to the spread of cholera. The increasing hydro-toxicity of underground water is another challenge to the disease spread. The lack of public awareness about the disease and their poor hygiene practices serve as a portal for the disease to spread to humans. There is a need to establish an effective surveillance system, increase public awareness, and implement the WHO-recommended plan for cholera control. This includes the provision of drugs and diagnostic kits to healthcare centres, the supply of clean water, and the development of a drainage system for sewage and rainwater.

## Introduction

Cholera is an acute diarrhoeal infection caused by ingestion of food or water contaminated with the bacterium *Vibrio cholerae* [1]. This illness most commonly presents with profuse watery diarrhoea described as “rice water stools” [2]. Vomiting, leg cramps, increased thirst, and restlessness are accompanying symptoms. Signs of dehydration like loss of skin elasticity and dry mucous membranes are also discernible in most patients [3].

To date, the world has seen 6 pandemics of cholera since the start of the nineteenth century. These pandemics have caused the deaths of millions of people all over the world. Currently, we are facing the 7th pandemic which started in South Asia in 1961 [4]. The most recent outbreaks in 2021 and 2022 have been reported in several countries on the Asian and African continents [5]. The causative agent behind these outbreaks has been the O1 serogroup of *V. cholera*e [4].

In Pakistan, cholera is an endemic disease with a threshold of a single laboratory-confirmed case to be declared an epidemic [6]. The most recent outbreak began in Sindh in 2022. The disease has made its way into Baluchistan and Punjab in the last few months [7]. A total of 290 confirmed cases of cholera had been reported in these provinces as of 27 May [6]. At the moment, the metropolitan city of Lahore, the capital of Punjab, is facing a full-frontal assault that has resulted in over 2000 children being hospitalized due to diarrhoea since April. A total of 25 lab-confirmed cases have been reported in Punjab, the majority of them being in Lahore, as of 27 May and this number is only expected to go up [6].

## Challenges and efforts

There are certain factors that precipitate the outbreak of cholera. Of these, the root cause is overpopulation due to which inhabitants are deprived of a healthy quality of life owing to a lack of proper sanitation, clean water supply, and housing conditions [8]. Lahore is the second most populous city in Pakistan with a population of over 7 million residing in an area of 1772 sq. km [9]. Administrative problems arise in managing such a heavily populated area such as the under-reporting of cases. This in turn leads to improper allocation of funds for health and associated domains.

Another problem that goes hand in hand with overpopulation is poor sanitary conditions. Lahore is ranked 2nd amongst the cities with a poor sanitation system with 42% of its population having no access to basic sanitation [10]. A large proportion of the population is inhabiting the slums which lack basic facilities. A recent survey indicated that 10% of this population uses open fields as make-shift toilets and the rest are provided with actual toilets of which 55% lack a flushing system [11].

The source of water in such settlements is rivers, streams, etc. The assessment of water quality indicated that 59 out of 60 samples were heavily contaminated with coliform organisms. Drinking water was also found to be unsafe [11]. What adds to this is the poor drainage system for rainwater which promotes the contamination of the water supply by sewage waste [12]. This is evidenced by the surge in the cases of cholera in rainfall seasons.

However, a recent spike was observed in the number of cases of cholera in May 2022, before the monsoon season. This is due to hydro-toxicity which is the toxification of underground water reserves by inorganic and organic wastes.-[13] The majority of the population uses underground water, and its contamination predisposes masses to water-borne diseases.

Another challenge is the lack of awareness which is the reason behind the deficit in preventative efforts on the public’s part. A survey indicated poor hygiene and hand washing practices in the majority of the population [14]. Additionally, knowledge about the source of drinking water and the risk of its possible contamination is lacking [14]. This problem is further exacerbated by social and economic constraints.

Another notable problem that should not be overlooked is the lack of proper surveillance and testing for the disease [7]. Mostly cholera is diagnosed upon clinical suspicion, but this diagnosis is confirmed only by microbiological stool cultures or by molecular studies such as PCR. Unfortunately, there is a lack of effort and resource allocation in this regard by the Pakistan government. This failure eventually translates into poor policy-making that further exacerbates the situation.

Still, some efforts are currently being undertaken to control this outbreak in Lahore by the Punjab Government. Government officials have talked about detection and diagnosis of cases being the foremost priority. Special counters are being set up in hospitals for the diagnosis of diarrhoea and cholera. The government is trying to ensure the availability of ORS, zinc, IV fluids, antibiotics and the chlorination of drinking water to make it safe for drinking [15]. A “Cholera response plan” has also been prepared which includes effective reporting of cases and their management, the involvement of the community through public awareness, and the use of oral cholera vaccine in highly endemic areas [6]. WHO has also taken steps to engage the Global Task Force on Cholera Control (GTFCC) to ensure the implementation of its preventative strategies [4]. Despite these efforts, the outbreak appears to be breaking out of control, which indicates the failure of the “Cholera response plan” by the government.

## Recommendations

In order to contain the cholera epidemic, efforts need to be made at an individual and community level by the public and the government. There needs to be a surveillance system to ensure reporting of cases, their management, and follow-up [16]. The WHO plan for cholera control can be used to address the disease outbreak. It should be ensured that the control strategies for the disease are being implemented and for this feedback is essential [2].

The first and foremost responsibility has to be borne by the Health department of Punjab. The cases can be managed at the hospitals if they are well equipped with trained personnel. The provision of diagnostic kits to the hospitals and the medications for management needs to be ensured. Vaccine production and its availability and proper administration in the hospitals should also be ensured for keeping the toll low in highly endemic areas [17]. Moreover, the stakeholders of large private sector hospitals should also be incentivized to take a small walk outside of the confines of for-profit health provision and play their part in sharing the burden in such catastrophic situations. Affordable emergency and testing services are something that they should volunteer to reliably ensure [18].

Of immense importance is the government’s responsibility to ensure a clean water supply. There is a dire need to set up sanitary latrines and waste disposal systems [19]. In order to prevent the contamination of water sources, the wastewater should be properly treated and disposed of. Measures should be taken for the drainage of rainwater as the monsoon is just around the corner.

Keeping in view the low literacy rates, the public should be educated about the disease, its symptoms, and prevention through awareness campaigns and media portals. Proper hygiene care should be advised in day-to-day activities like food preparation and funeral practices, as the body fluids of the deceased can be a source of cholera transmission [20]. An important role in health education has to be played by every individual stakeholder including general practitioners and policymakers alike. This would prove to be not only the first line of defence against any future outbreaks, but also the most stalwart one. On a mass level, the efforts by the government to eradicate the root problems of overpopulation and economic instability will help prevent disease outbreaks by improving the living condition of the masses.

## Data Availability

None.
